# A new binuclear Ni(II) complex, an effective A^3^-coupling catalyst in solvent-free condition

**DOI:** 10.1016/j.heliyon.2023.e17743

**Published:** 2023-07-01

**Authors:** Ayda Sheykhi, Ali Akbar Khandar, Jan Janczak, Mojtaba Amini

**Affiliations:** a Department of Inorganic Chemistry, Faculty of Chemistry, University of Tabriz, P.O. Box 5166616471, Tabriz, Iran; b Institute of Low Temperature and Structure Research, Polish Academy of Sciences, Okólna 2 str. 50-422, Wrocław, Poland

**Keywords:** Binuclear, Nickel, A^3^-coupling, Solvent-free

## Abstract

A newly binuclear nickel(II) complex, [Ni_2_(en)_4_(ox)](ClO_4_)_2_ (**1**) (where en = ethylenediamine, and ox = oxalate), has been isolated from a reaction of NiCl_2_·6H_2_O, ethylenediamine, ammonium oxalate and sodium perchlorate in water and its crystal structure has been determined by X-ray crystallography and infra-red techniques. Compound **1** was successfully employed to promote the one-pot reaction of aldehydes, amines and acetylenes for the construction of corresponding propargylamines under solvent-free media with fine yields. Further studies reveal this catalytic system can be refreshed and used again in five runs.

## 1 Introduction

The oxalate anion as one of the simplest bridging ligands plays a structural role in designing homo- and hetero di-, oligo- and poly-nuclear complexes which can expand in one-, two- or three-dimensional structures [1,2]. Oxalate-based dinuclear complexes consist of a wide range of transition metals such as copper [3,4], manganese [5], zinc [6,7], iron [2], cobalt [8], and zirconium [9]. Among these, several binuclear μ-oxalato Ni(II) complexes were reported previously and their properties were investigated [[10], [11], [12], [13], [14]]. These types of complexes due to the oxalato ligand ability in the establishing the magnetically and structural relationships between complex species have attracted much attention within inorganic chemistry [12,15]. Designing and preparation of inorganic-organic mix catalysts are of special significance in the preparation of synthetically and industrially made compounds [4,16]. A countless complexes based on transition metals have been reported as catalytic systems in plentiful organic transformations reactions [[17], [18], [19], [20], [21]]. Industrially, after cobalt, nickel is second as the most commonly used transition metal in catalytic transformations [22]. Frequent efforts are being made in the industry to replace second or third-row transition elements (e.g. Pd and Rh) with low-cost first-row transition metals (e.g. Ni). However, improving the efficiency of these metals is one of the challenges facing researchers [18,[23], [24], [25]]. On the other side, after iron, titanium, and zirconium, Ni is the fourth-ranked transition metal on our planet and so that, designing routes for developing effective applications of nickel compounds for catalytic systems is desirable [26].

Recently, propargylic compounds, particularly propargylamines, have gained considerable attention as valuable units for creating heterocyclic compounds. They are seen as ideal starting materials for a vast variety of medicinally and biologically important scaffold structures, due to their easy accessibility and possession of two functional groups (a triple bond and an amino group) that can undergo further modifications [27,28]. For example, silver(I) acetate-catalyzed preparation of oxazolidinones from terminal and internal propargylamines [29], synthesis of biologically promising quinolones by the cyclization of propargylamine derivatives in the presence of catalytic amount of CuCl [30], or synthesis of highly substituted pyrroles via cycloaddition of *N*-propargylamines and C–C double bonds [31].

Furthermore, some propargylamines are important therapeutic drug species that show many remarkable properties including neurodegenerative diseases therapy [32,33]. Previously, some less attractive routes with hard operational and harsh reaction conditions such as organo-lithium and -zinc reagents and Grignard reagents have been utilized [[34], [35], [36]]. Recently, transition metal-catalyzed aldehyde-amine-alkyne coupling (A^3^-coupling) has received more attention because of its one-pot fabrication, atomic economy process, and high selectivity reaction [[37], [38], [39]].

In order to look for inexpensive catalysts for green sustainable catalytic syntheses of propargylamines, this study aimed to describe the preparation and crystalline structure of a novel dinickel μ-oxalato-bridged complex as a catalytic system for the A^3^-coupling transformations of aldehydes, amines and terminal alkynes. [Ni_2_(en)_4_(ox)](ClO_4_)_2_ was synthesized with a facile approach and inexpensive precursors and applied as an effective green catalyst for the synthesis of propargylamines with a solventless process.

## 2 Experimental section

### 2.1 Chemicals and methods

All materials were provided from mercantile reagent sources and utilized without extra refinement. Nickel(II) chloride hexahydrate (99%), ethylenediamine (99%), ammonium oxalate (99.5%), and sodium perchlorate were purchased from Sigma Aldrich and utilized for the synthesis of [Ni_2_(en)_4_(ox)](ClO_4_)_2_. Phenylacetylene (98%), propargyl alcohol (99%), morpholine (99%), piperidine (99%), benzaldehyde (99%), 4-methylbenzaldehyde (97%), furfural (98%), 1-methylpyrrole-2-carboxaldehyde (98%) were purchased from Merck and used for the A^3^-coupling reaction. All solvents used in this work, consist of N,N-dimethylformamide (DMF), ethanol, methanol, n-hexane, ethyl acetate, toluene, tetrahydrofuran (THF), acetonitrile dichloromethane and dimethyl sulfoxide (DMSO), were of analytical grade. An ATR-FTIR PerkinElmer (model: Spectromrx) apparatus was employed for the determination of IR absorption peaks of complex **1** after and before use it as an A^3^-coupling reaction catalyst. UV–vis absorption spectrum of the fresh and reused catalyst was recorded by Shimadzu UV-160A spectrophotometer. Energy dispersive X-ray spectrometry (EDS) elemental analysis was performed by a TESCAN (model: MIRA II, detector: SAMX) FESEM apparatus which has an EDS facility attachment.

### 2.2 Synthesis of [Ni2(en)4(ox)](ClO4)2 (1)

Ethylenediamine (0.2 mL, 3.0 mmol) in H_2_O (12 mL) was deaerated by bubbling Nitrogen gas inside the solution for 10 min, and nickel(II) chloride hexahydrate (0.71 g, 3.0 mmol) in water (5 mL) and ammonium oxalate in water (5 mL, 0.3 M) was added. The N_2_ flow was maintained for 5 min. Sodium perchlorate solution (1 mL, 1 M) bubbled with N_2_ and was added to the mentioned mixture and then was held in a crystallizer at ambient conditions. About 3 days later, the purple crystals were separated and washed with methanol. Yield about 76% based on nickel. EDS Elemental Anal. Calcd for C_10_H_32_N_8_Ni_2_O_12_Cl_2_ (%): Ni, 18.21; N, 17.38; C, 18.63; Cl, 11.00; O, 29.78. Found (%): Ni, 18.08; N, 17.31; C, 18.54; Cl, 11.13; O, 29.93.

Caution: Perchlorate salts should be handled with great care because perchlorates are dangerous and can explode when heated.

### 2.3 Typical catalytic process for one-pot three-component A3-coupling

In general process for the synthesis of propargylamines, a mixture including terminal alkyne (1.0 mmol), secondary amine (1.1 mmol), aldehyde (1.2 mmol), and complex **1** (0.9 mol%) was reacted in an open-air atmosphere at 100 °C for the desired reaction period. Thin Layer Chromatography (TLC) was employed to check and control the end-point of the reaction. After termination of the reaction, the reaction vessel was left to cool down to ambient temperature and corresponding propargylamine was extracted by the addition of water (5 mL) and ethyl acetate (20 mL in two-step) to a separatory funnel. After standing for 10 min, the mixture separated into a colorless lower layer and a clear yellow upper layer. The upper organic layer was collected. After extraction, the organic solution was dried over anhydrous calcium chloride, and the solvent was evaporated with a rotary evaporator at 60 °C to give the corresponding propargylamine without the need for further purification.

### 2.4 Reusability

After completion of the A^3^-coupling reaction between phenylacetylene, morpholine, and benzaldehyde in the presence of complex **1** (20 mg), the corresponding product was extracted according to the above explained process. Then, ethyl acetate (about 10–15 mL) was added to the aqueous solution (upper layer in the separatory funnel) and the catalyst precipitated from solution. The catalyst was then filtered, washed twice with ethyl acetate (∼5 mL) and then diethyl ether (∼5 mL), dried at 50 °C for 60 min, and reused for a new cycle of the A^3^-coupling reaction of phenylacetylene, morpholine, and benzaldehyde with no need for further additions of catalyst **1** at 100 °C for 5 h. After five runs the catalyst was collected and used for the analysis of IR and XRD.

## 3 Results and discussion

### 3.1 Characterization

**Fig. 1:**
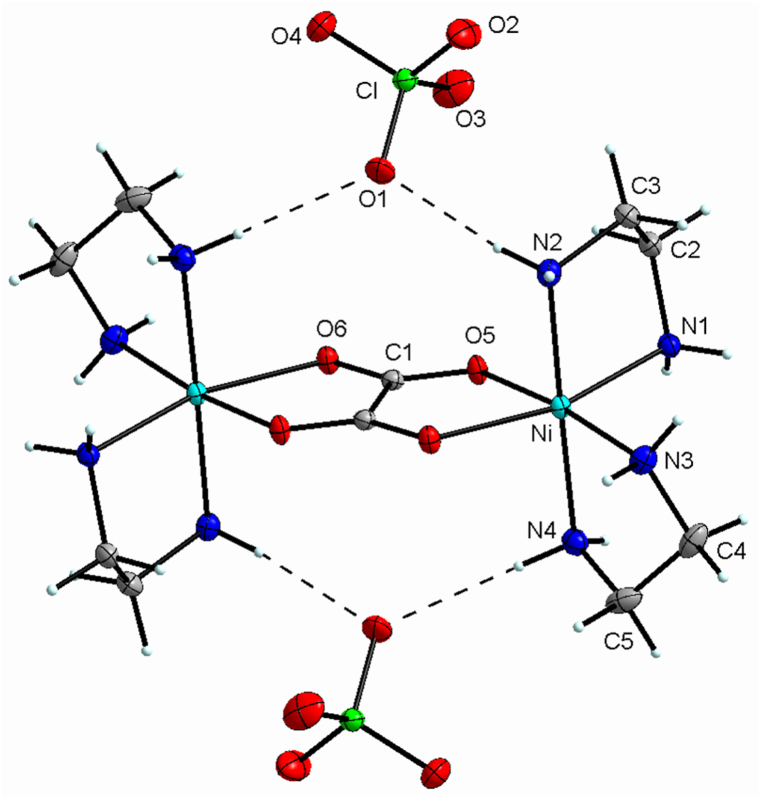
ORTEP drawing for complex **1**. Anisotropic displacement ellipsoids are at the 50% possibility level. H atoms represent the circle with arbitrary radii.

The [Ni_2_(en)_4_(ox)](ClO_4_)_2_ was produced by the reaction of nickel(II) chloride with ethylenediamine, sodium perchlorate, and ammonium oxalate in aqueous solution and the crystal structure of compound **1** structurally characterized by the single-crystal X-ray studies. An ORTEP-type view of the complex is presented in Fig. 1, and Crystal data and experimental parameters for complex **1** are exhibited in Table 1. This complex formed into crystals with a monoclinic crystal system with *P*2_1_/*n* centrosymmetric space group. The structure of **1** is made up of two nickel (II) centers, and each Ni (II) atom has a similar stereochemistry. Each of the six-coordinate octahedral geometry Ni (II) ions is surrounded by two ethylenediamine ligands and half of the oxalate bridge. In addition, two perchlorate anions play a counter-ion role. The bond distances of oxygens in oxalato ligand and nickel centers are in the range of 2.0809(12) and 2.1005(12) Å (with a mean bond length of 2.0907 Å), which are similar to formerly reported same compound bond lengths [[12], [13], [14]]. Ni–N bond lengths are from 2.0810(16) to 2.1058(15) Å (with an average bond longitude of 2.0971 Å). Also, the bond distance mean of C(en)-N(en), C(en)-C(en), C(ox)-O(ox), C(ox)-C(ox) are 1.478, 1.512, 1.254, 1.554 Å. Furthermore, N–Ni–N bond angles are in the range of 82.38(1) and 177.74(1)°, and O–Ni–O bond angle is 80.26(1)°.

**Table 1:** Summary of crystallographic data for the complex **1**.

	[Ni_2_(en)_4_(ox)](ClO_4_)_2_
Formula	C_10_H_32_N_8_Ni_2_O_4_·2(ClO_4_)
FW (g.mol^−1^)	644.75
Temp. [K]	100(1)
λ [Å]	0.71073
Cryst syst	Monoclinic
Space group	*P*2_1_/*n*
a [Å]	7.0920(4)
b [Å]	20.6153(9)
c [Å]	8.7653(5)
β [deg]	108.346(7)
V [Å^3^]	1216.38(12)
Z, ρ_calc_ [g cm^−3^]	2
μ [mm^−1^]	1.838
F(000)	688
Refl. total Refl. unique Refl. observed [*I* > *2σ(I)*]	13,196 2752 2363
R_int_ Abs. corr.	0.0341 Multi-Scan
Min., max. Transmission factors	0.8212/1.0000
*R1 [I* > *2σ(I)]*	0.0254
wR_2_ (all data) S Δρ_max_, Δρ_min_ (eÅ^−3^)	0.0557 1.001 0.344, −0.421

**Fig. 2:**
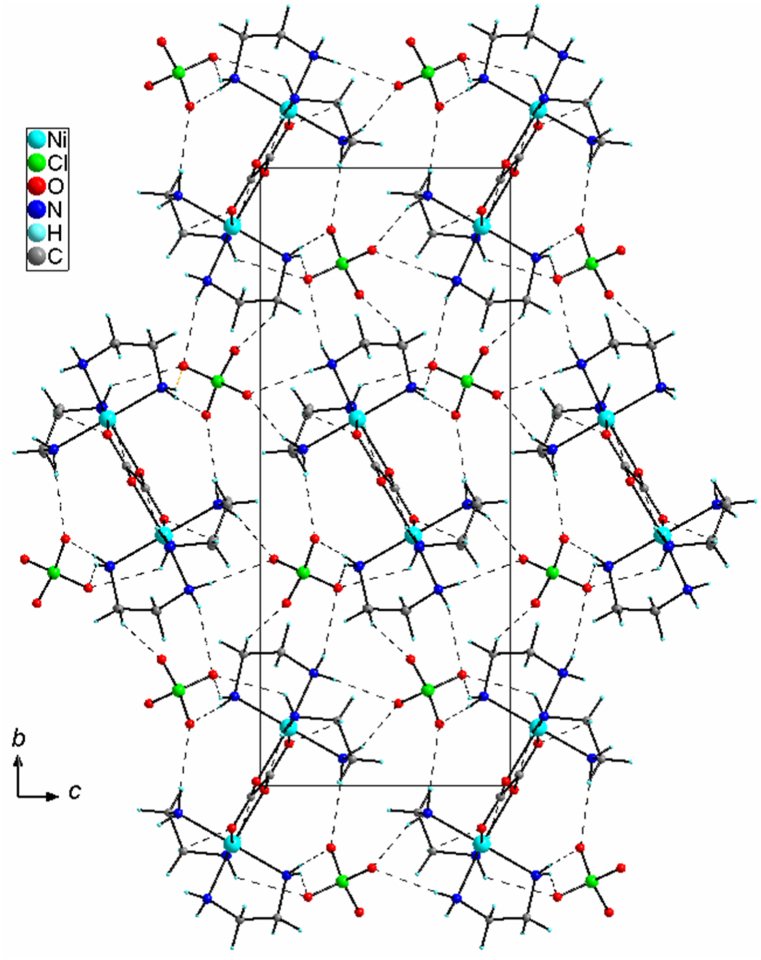
Crystal packing of **1** showing a three-dimensional network of hydrogen bonds.

The charge of the [Ni_2_(en)_4_(ox)]^2+^ cation is balanced by two perchlorate anions that act as hydrogen bond acceptors with the amino groups of ethylenediamine. In the crystal, all H atoms of NH_2_ groups of coordinated ethylenediamine molecules are involved in the N–H⋯O hydrogen bonds (Table 2) making a three-dimensional hydrogen bonding network (Fig. 2).

**Table 2:** Hydrogen-bond geometry (Å, °).

*D—H···A*	*D—H*	*H···A*	*D···A*	*D—H···A*
N1–H1A···O4^*i*^	0.90 (2)	2.36 (2)	3.210 (2)	157 (2)
N1–H1B⋯O3^*ii*^	0.89 (2)	2.44 (2)	3.234 (2)	150 (2)
C3–H31⋯O2^*ii*^	0.99	2.56	3.498 (2)	158
N2–H2A···O3^*iii*^	0.84 (2)	2.49 (2)	3.221 (2)	145 (2)
N2–H2B⋯O1	0.85 (2)	2.22 (2)	3.065 (2)	169 (2)
N3–H3A···O3^*iii*^	0.83 (2)	2.57 (2)	3.161 (2)	130 (2)
N3–H3B⋯O5^*iii*^	0.87 (2)	2.63 (2)	3.179 (2)	122 (2)
N3–H3B⋯O6^*iii*^	0.87 (2)	2.29 (2)	3.137 (2)	165 (2)
N4–H4A···O4^*i*^	0.87 (2)	2.48 (2)	3.322 (2)	163 (2)
C4–H41⋯O5^*iii*^	0.99	2.56	3.251 (2)	126
N4–H4B⋯O1^*iv*^	0.89 (2)	2.24 (2)	3.130 (2)	174 (2)

Symmetry codes: (*i*) x, y, z − 1; (*ii*) x + 1/2, −y + 3/2, z − 1/2; (*iii*) x + 1, y, z; (*iv*) −x + 1, −y + 1, −z + 1.

### 3.2 Catalytic studies

Having in hand, the [Ni_2_(en)_4_(ox)](ClO_4_)_2_ catalyst, we began the initial examination of its catalytic activity. As the model reaction, we selected a three-component reaction of phenylacetylene, morpholine, and benzaldehyde using complex **1** as a catalyst in various solvents (Table 3). The loading of the catalyst was first examined with the reaction completed at 70 °C for 5 h. When the reaction was performed in the absence of complex **1** in solvent-free conditions at 100 °C, no product was achieved even after 10 h (entry 1). The results demonstrate that with 0.9 mol% of catalyst, the maximum yield was achieved after 5 h (95%, entry 23). Nevertheless, decreasing the catalytic amount from 0.9 to 0.3 mol% conversion was decreased from 95 to 64% (entries 2–4). Raising the catalyst amount to 1.1 mol% did not increase the yield of the propargylamine product (entry 5). Furthermore, in the presence of 0.9 mol% NiCl_2_·2H_2_O and NiC_2_O_4_·2H_2_O, 68% and 57% yield of the propargylamine was achieved, respectively (entry 26 and 27). To find the most optimal solvent, the reaction was carried out in different solvents, for example, water, N,N-dimethylformamide, ethanol, methanol, n-hexane, ethyl acetate, toluene, tetrahydrofuran, acetonitrile dichloromethane and dimethyl sulfoxide (entries 6–16). When the reactions were carried out by using solvent, a low yield of propargylamine product was obtained. The outcomes revealed that the presence of a solvent is detrimental for the preparation of the product, and the reaction completion was performed with a solventless process. Then the optimization continues to obtain the appropriate reaction temperature required for the synthesis of the related products. For this aim, a series of experiments were utilized in different reaction temperatures. The results of these tests display the key impact of temperature in the catalytic reaction for the preparation of propargylamines. So, when the reaction was done at ambient temperature only 17% product was achieved, and the increasing temperature to 100 °C, the reaction yield raised gradually to 95% (entries 17–21). Nonetheless, a further increase in the reaction temperature to 110 °C, did not have a positive impact on the yield of the propargylamine (entry 22). Moreover, to find the optimal reaction conditions, we try to find the effect of the reaction period on the yield of the model reaction (entries 23–25). The highest yield was recorded when the reaction was performed for 5 h. In order to further study the effect of different reaction conditions on the A^3^-coupling reaction, the results of the synthesis of 4-(1,3-diphenylprop-2-yn-1-yl)morpholine via benzaldehyde, morpholine and phenylacetylene at various temperatures, reaction times, and catalyst dosages were summarized in Figs. S1–S3.

**Table 3:** The effect of different reaction conditions on the three-component A^3^-coupling.^a^

Entry	Cat.	Cat. loading (mol%)	Solvent	Temp.^b^ (°C)	Time (h)	Yield^c^ (%)
1	–	–	None	100	10	6
2	**1**	0.3	None	100	5	64
3	**1**	0.5	None	100	5	79
4	**1**	0.7	None	100	5	90
5	**1**	1.1	None	100	5	95
6	**1**	0.9	H_2_O	Reflux	5	52
7	**1**	0.9	DMF	100	5	48
8	**1**	0.9	EtOH	Reflux	5	35
9	**1**	0.9	MeOH	Reflux	5	29
10	**1**	0.9	n-hexane	Reflux	5	41
11	**1**	0.9	MeCOOEt	Reflux	5	19
12	**1**	0.9	Toluene	100	5	61
13	**1**	0.9	THF	Reflux	5	38
14	**1**	0.9	MeCN	Reflux	5	59
15	**1**	0.9	CH_2_Cl_2_	Reflux	5	26
16	**1**	0.9	DMSO	100	5	56
17	**1**	0.9	None	r.t.	5	17
18	**1**	0.9	None	40	5	26
19	**1**	0.9	None	60	5	42
20	**1**	0.9	None	80	5	71
21	**1**	0.9	None	100	5	95
22	**1**	0.9	None	110	5	94
23	**1**	0.9	None	100	1	58
24	**1**	0.9	None	100	3	81
25	**1**	0.9	None	100	7	95
26	NiCl_2_·6H_2_O	0.9	None	100	5	68
27	NiC_2_O_4_·2H_2_O	0.9	None	100	5	57

a Reaction conditions: phenylacetylene (1.0 mmol), morpholine (1.1 mmol), benzaldehyde (1.2 mmol), solvent (2.0 mL).

b The reflux temperature was 4–6 °C more than solvent b.p.

c Isolated yield.

Under the optimized experimental conditions, various aldehydes, secondary amines, and terminal alkynes were reacted with complex **1** without using solvent at 100 °C to generate the related products and the results were collected in Table 4. As can be observed from Table 4 results, catalyst **1** has a wide performance area, and amines involved morpholine and piperidine, aldehydes consisting of benzaldehyde, 4-methylbenzaldehyde, 4-chlorobenzaldehyde, 2-chlorobenzaldehyde, 4-nitrobenzaldehyde, 4-methoxybenzaldehyde, furfural, and 1-methylpyrrole-2-carboxaldehyde, and terminal acetylenes included phenylacetylene, 4-methylphenylacetylene, and propargyl alcohol reacted efficiently to conclude in the synthesis of propargylic amines in good to excellent yields (70–97%). In addition, the activity of piperidine in the reactions with aldehydes and alkynes is higher than that of morpholine. Furthermore, It seems the methyl group in 1-methylpyrrole-2-carboxaldehyde and 2-chloro group in 2-chlorobenzaldehyde because of the steric hindrance effects, displayed a slightly decreasing in the yield of corresponding products (entries 12, 13, 19 and 20).

**Table 4:** A^3^-coupling reaction of various terminal acetylenes, amines and aldehydes.^a^

/		Aldehyde	Amine	Alkyne	Yield^b^ (%)
1	1				95
2	2				97
3	3				93
4	4				94
5	5				88
6	6				90
7	7				86
8		0			92
9		1			94
10		2			77
11		3			76
12		4			70
13		5			73
14		6			82
15		7			85
16		8			85
17		9			89
18	8				93
19	9				86
20	10				83

a Reaction conditions: alkyne (1.0 mmol), amine (1.1 mmol), aldehyde (1.2 mmol), cat. (0.9 mol%), solvent-free, 100 °C and 5 h.

b Isolated yield.

Overall, the presence of various substituents in benzaldehyde leads to a decrease in the yield of the reaction (entries 5–7 and 10–17). However, the impact of electron-withdrawing groups on reducing yield is greater (entries 10–15). Also, the presence of a methyl group on the phenyl ring of phenylacetylene results in a slight decrease in the reaction yields (entries 8 and 9).

### 3.3 Reusability

**Fig. 3:**
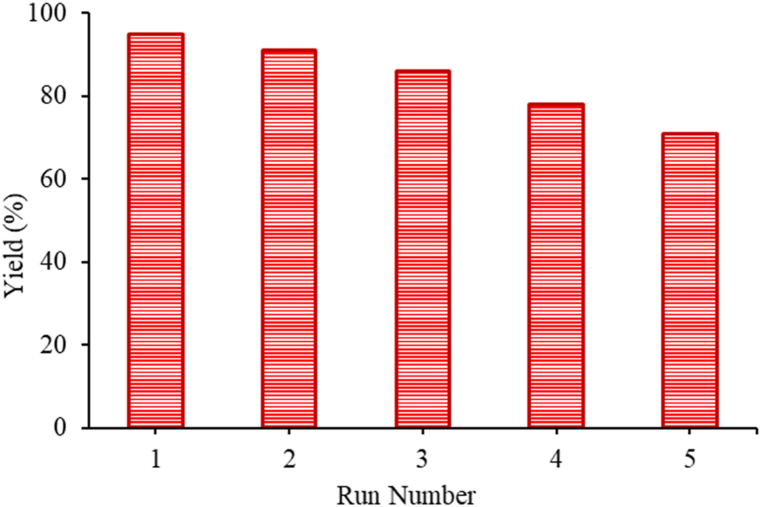
Recycling studies of catalyst **1** in the A^3^-coupling reaction.

For catalytic heterogeneous systems, it is significant to investigate the recovery and reusability of the catalyst. The recycling of complex **1** was studied at the reaction completion. Ethyl acetate was employed to extract the product, and the solid phase was collected by filtration. The collected catalyst was washed several times with ethyl acetate and then diethyl ether and dried in an oven. The recovered catalyst was then used in the second cycle without further treatment. Catalyst **1** (20 mg) could be recycled in five continuous cycles with a slight loss of activity (Fig. 3).

**Fig. 4:**
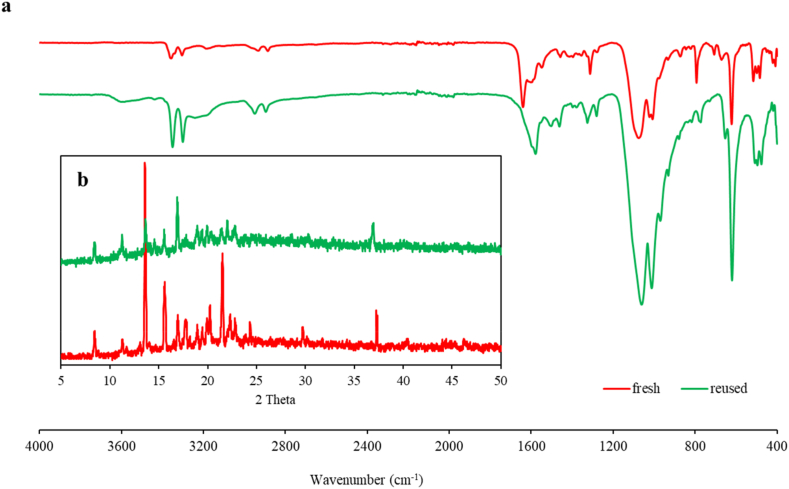
a) FTIR spectra and b) XRD patterns of fresh (red lines) and reused (green lines) complex **1**. (For interpretation of the references to color in this figure legend, the reader is referred to the Web version of this article.)

Fig. 4 shows the ATR-FTIR spectra and XRD patterns of catalyst **1** as fresh and after the A^3^-coupling reaction for the synthesis of propargylamine. The absorption peak at 410 cm^−1^ in FTIR spectra is due to the Ni–O bonds. The peak at 488.9 cm^−1^ is assigned to the characteristic vibration bands of Ni (II)–O and C–C. The band 600-700 cm^−1^ is due to Ni–O stretching vibration. The peak at 1650 is corresponded to the ν_asym_(O–C–O) [40]. The two bands at 3357 and 3301 cm^−1^ are attributed to the asymmetric and symmetric stretch of the H–N–H groups. The absorption peaks at 2929 and 2885 cm^−1^ are due to the asymmetric and symmetric H–C–H stretching vibration. The band at 1597 cm^−1^ is attributed to H–N–H bending vibration. The comparison of fresh and reused spectra of complex **1** demonstrated no major changes, indicating good stability of catalyst **1** after several cycles of reusing.

To confirm the stability of the catalyst **1** during the coupling reaction, the XRD patterns of the fresh and reused catalyst after five steps were obtained. As shown in Fig. 4b, the XRD patterns of catalyst after recycling match well with fresh catalyst, indicating a good stability of the catalyst.

**Scheme 1:**
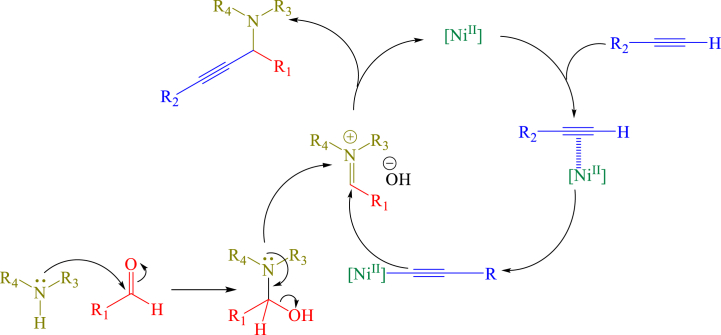
Proposed mechanism for A^3^-coupling reaction by catalyst **1**.

The synthesis of propargylamine via the A^3^-coupling proceeded smoothly, giving the related propargylamine by deprotonation of the terminal acetylenes. A possible mechanism involving deprotonation of alkyne by Ni^2+^ species was proposed to give a nickel acetylide intermediate. Then, the nickel acetylide intermediate generated and reacted with the iminium ion formed from aldehydes and amines to give the corresponding propargylamine (Scheme 1).

In order to display the catalytic efficiency of compound **1**, we compared the catalytic behavior of [Ni_2_(en)_4_(ox)](ClO_4_)_2_ with some formerly studied nickel-containing compounds in the catalytic synthesis of propargylamines from aldehydes, amines and acetylenes. Under the conditions pronounced in Table 5, the present catalytic system in some cases is more efficient than other protocols, for example, solvent-free condition (entries 2 and 3), low reaction time (entries 2 and 3), lower catalyst dosages (entries 2–4), without needing to reaction under inert atmosphere (entries 2 and 3), simple catalyst preparation protocol (entries 1, 2 and 4).

**Table 5:** A^3^-coupling reaction with different Ni-containing compounds.

Entry	Catalyst (amount)	Condition: substrates/solvent/temp./time	Yield (%)	Ref.
1	NiL_2_ (2 mol%)	Benzaldehyde, pyrrolidine, phenylacetylene/solvent-free/85 °C/2 h	86	[41]
2	NiCl_2_ (5 mol%), argon atmosphere	Benzaldehyde, piperidine, phenylacetylene/PhMe/111 °C/8 h	95	[42]
3	PANF_TPA_@NiCl_2_^a^ (5 mol%), N_2_ atmosphere	Benzaldehyde, pyrrolidine, phenylacetylene/CH_3_CN/reflux/6 h	85	[43]
4	Ni–Y zeolite (20 mg)	Cyclohexyl, pyrrolidine, phenylacetylene/solvent-free/80 °C/4 h	97	[44]
5	[Ni_2_(en)_4_(ox)Cl_2_]·2(ClO_4_) (0.9 mol%)	Benzaldehyde, morpholine, phenylacetylene/solvent-free/100 °C/5 h	95	Present work

a PANF = polyacrylonitrile fiber, TPA = tetraethylene pentaamine.

## 4 Conclusions

A dinuclear Ni(II) complex was prepared by a reaction of nickel(II) chloride with ethylenediamine and ammonium oxalate in a neutral medium. Characterization of this compound was carried out by single-crystal X-ray crystallography and FTIR analysis. This compound displayed high activity catalytic performance in the A^3^-coupling reaction of acetylenes, amines and aldehydes for the synthesis of propargylamines in solvent-free conditions. Catalyst **1** exhibited relatively high stability activity in comparison to other similar complexes referring to recoverability and reusability results and IR spectra before and after reuse to compound **1**.

## Author contribution statement

Ayda Sheykhi: Performed the experiments; Wrote the paper.

Ali Akbar Khandar: Conceived and designed the experiments.

Jan Janczak: Contributed reagents, materials, analysis tools or data.

Mojtaba Amini: Conceived and designed the experiments; Analyzed and interpreted the data.

## Data availability statement

Data included in article/supp. material/referenced in article.

## Declaration of competing interest

The authors declare that they have no known competing financial interests or personal relationships that could have appeared to influence the work reported in this paper.
