# Supplementary material for: A new binuclear Ni(II) complex, an effective A3-coupling catalyst in solvent-free condition
Source: Heliyon. 2023 Jul 1;9(7):e17743. doi: 10.1016/j.heliyon.2023.e17743 (PMC10395144; doi:10.1016/j.heliyon.2023.e17743)

checkCIF/PLATON report

Structure factors have been supplied for datablock(s) H8_100

THIS REPORT IS FOR GUIDANCE ONLY. IF USED AS PART OF A REVIEW PROCEDURE FOR PUBLICATION, IT SHOULD NOT REPLACE THE EXPERTISE OF AN EXPERIENCED CRYSTALLOGRAPHIC REFEREE.

No syntax errors found. CIF dictionary Interpreting this report

Datablock: H8_100

Bond precision:	C-C = 0.0030 A	Wavelength=0.71073	
Cell:	a=7.0920(4)	b=20.6153(9)	c=8.7653(5)
	alpha=90	beta=108.346(7)	gamma=90
Temperature:	100 K		

	Calculated	Reported
Volume	1216.38(12)	1216.38(12)
Space group	P 21/n	P 21/n
Hall group	-P 2yn	-P 2yn
Moiety formula	C10 H32 N8 Ni2 O4, 2(Cl O4)	C10 H32 N8 Ni2 O4, 2(Cl O4)
Sum formula	C10 H32 Cl2 N8 Ni2 O12	C10 H32 Cl2 N8 Ni2 O12
Mr	644.72	644.75
Dx,g cm-3	1.760	1.760
Z	2	2
Mu (mm-1)	1.838	1.838
F000	668.0	668.0
F000'	670.22	
h,k,lmax	9,26,11	9,26,11
Nref	2802	2752
Tmin,Tmax	0.614,0.793	0.821,1.000
Tmin'	0.583	

Correction method= # Reported T Limits: Tmin=0.821 Tmax=1.000
AbsCorr = MULTI-SCAN

Data completeness= 0.982 Theta(max)= 27.497

R(reflections)= 0.0254(2363) wR2(reflections)= 0.0557(2752)

S = 1.001 Npar= 178

The following ALERTS were generated. Each ALERT has the format
test-name_ALERT_alert-type_alert-level.
Click on the hyperlinks for more details of the test.

Alert level G

```
PLAT480_ALERT_4_G Long H...A H-Bond Reported H3B      ..O5       .       2.63 Ang.
PLAT794_ALERT_5_G Tentative Bond Valency for Ni        (II)      .       1.98 Info
PLAT910_ALERT_3_G Missing # of FCF Reflection(s) Below Theta(Min).          1 Note
PLAT912_ALERT_4_G Missing # of FCF Reflections Above STh/L=  0.600         49 Note
PLAT941_ALERT_3_G Average HKL Measurement Multiplicity ...........        4.8 Low
PLAT961_ALERT_5_G Dataset Contains no Negative Intensities .......     Please Check
PLAT965_ALERT_2_G The SHELXL WEIGHT Optimisation has not Converged     Please Check
PLAT967_ALERT_5_G Note: Two-Theta Cutoff Value in Embedded .res ..       55.0 Degree
PLAT978_ALERT_2_G Number C-C Bonds with Positive Residual Density.          1 Info
```

0 **ALERT level A** = Most likely a serious problem - resolve or explain
0 **ALERT level B** = A potentially serious problem, consider carefully
0 **ALERT level C** = Check. Ensure it is not caused by an omission or oversight
9 **ALERT level G** = General information/check it is not something unexpected

0 ALERT type 1 CIF construction/syntax error, inconsistent or missing data
2 ALERT type 2 Indicator that the structure model may be wrong or deficient
2 ALERT type 3 Indicator that the structure quality may be low
2 ALERT type 4 Improvement, methodology, query or suggestion
3 ALERT type 5 Informative message, check

It is advisable to attempt to resolve as many as possible of the alerts in all categories. Often the minor alerts point to easily fixed oversights, errors and omissions in your CIF or refinement strategy, so attention to these fine details can be worthwhile. In order to resolve some of the more serious problems it may be necessary to carry out additional measurements or structure refinements. However, the purpose of your study may justify the reported deviations and the more serious of these should normally be commented upon in the discussion or experimental section of a paper or in the "special_details" fields of the CIF. checkCIF was carefully designed to identify outliers and unusual parameters, but every test has its limitations and alerts that are not important in a particular case may appear. Conversely, the absence of alerts does not guarantee there are no aspects of the results needing attention. It is up to the individual to critically assess their own results and, if necessary, seek expert advice.

Publication of your CIF in IUCr journals

A basic structural check has been run on your CIF. These basic checks will be run on all CIFs submitted for publication in IUCr journals (*Acta Crystallographica*, *Journal of Applied Crystallography*, *Journal of Synchrotron Radiation*); however, if you intend to submit to *Acta Crystallographica Section C* or *E* or *IUCrData*, you should make sure that full publication checks are run on the final version of your CIF prior to submission.

Publication of your CIF in other journals

Please refer to the *Notes for Authors* of the relevant journal for any special instructions relating to CIF submission.

PLATON version of 12/09/2022; check.def file version of 09/08/2022

Datablock H8_100 - ellipsoid plot

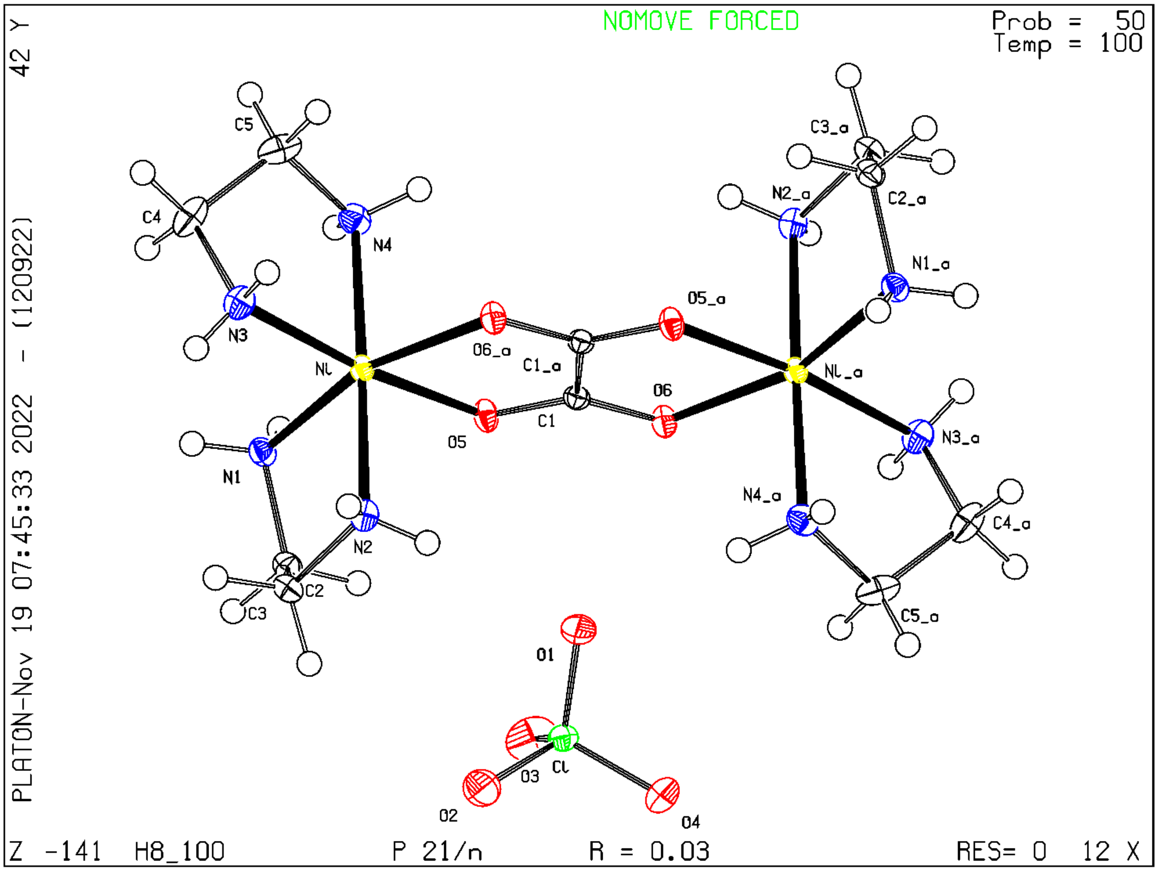

Supplement: Multimedia component 2 [file mmc2.pdf]
