# Supplementary material for: A new binuclear Ni(II) complex, an effective A3-coupling catalyst in solvent-free condition
Source: Heliyon. 2023 Jul 1;9(7):e17743. doi: 10.1016/j.heliyon.2023.e17743 (PMC10395144; doi:10.1016/j.heliyon.2023.e17743)
Supplement: Multimedia component 3 [file mmc3.docx]

**A new binuclear Ni(II) complex, an effective A^3^-coupling catalyst in solvent-free condition**

Ayda Sheykhi^a^, Ali Akbar Khandar^*a^, Jan Janczak^b^, Mojtaba Amini^*a^

*^a^ Department of Inorganic Chemistry, Faculty of Chemistry, University of Tabriz, P.O. Box 5166616471, Tabriz, Iran*

*^b^ Institute of Low Temperature and Structure Research, Polish Academy of Sciences, Okólna 2 str. 50-422 Wrocław, Poland*

**Characterization**

**Single crystal X-ray data collection**

X-ray intensity data for the [Ni_2_(en)_4_(ox)](ClO_4_)_2_ crystal (**1)** were collected using graphite monochromatic MoKα radiation on a four-circle κ geometry KUMA KM-4 diffractometer with a two-dimensional area CCD detector at RT (295 K) and LT (100 K) temperatures. Between the RT and LT no structural phase transitions were observed, therefore full data measurement for structural analysis was performed for these crystals at 100 K. Data collections were made using the CrysAlis CCD program [1]. Integration, scaling of the reflections, correction for Lorenz and polarisation effects and absorption corrections were performed using the CrysAlis Red program [1]. The structure was solved by the direct methods using SHELXT-2014/7 [2] and refined using SHELXL-2018/3 program [3]. The positions of hydrogen atoms (NH_2_) involving in the hydrogen bonds were located in difference Fourier maps and were refined with U_iso_=1.5Ueq of N joined H, whereas H atoms joined to carbon atoms were introduced in their geometrical positions and treated as rigid. The final difference Fourier maps showed no peaks of chemical significance. Details of the data collection parameters, crystallographic data and final agreement parameters are collected in Table 1. Visualizations of the structures were made with the Diamond 3.0 program [4].

The CIF file of crystal structure complex, [Ni_2_(en)_4_(ox)](ClO_4_)_2_ has been deposited with the CCDC, No. 2221599 This data can be obtained free of charge via http://www.ccdc.cam.ac.uk/conts/retrieving.html, or from the Cambridge Crystallographic Data Centre, 12 Union Road, Cambridge CB2 1EZ, UK; fax: (+44) 1223-336-033; or e-mail: [deposit@ccdc.cam.ac.uk](mailto:deposit@ccdc.cam.ac.uk)

**Investigation of various reaction conditions on the A^3^-coupling reaction of benzaldehyde, morpholine and phenylacetylene**

Fig. S1. Influences of loading of various catalysts and temperature for the synthesis of 4-(1,3-diphenylprop-2-yn-1-yl)morpholine

Fig. S2. Effects of various catalysts, temperature and duration of reaction for the synthesis of 4-(1,3-diphenylprop-2-yn-1-yl)morpholine

Fig. S3. Effects of temperature and solvent for the synthesis of 4-(1,3-diphenylprop-2-yn-1-yl)morpholine

**^1^H NMR Analysis for propargylamines (Table 4)**

Entry-1: 4-(1,3-diphenylprop-2-yn-1-yl)morpholine:

^1^H NMR (CDCl_3_, ppm): δ 7.66-7.63 (m, 2H), 7.54-7.51 (m, 2H), 7.38-7.31 (m, 6H), 4.82 (s, 1H), 3.77-3.73 (m, 4H), 2.66-2.64 (m, 4H)

Entry-2: 1-(1,3-diphenylprop-2-yn-1-yl)piperidine:

^1^H NMR (CDCl_3_, ppm): δ 7.67-7.64 (m, 2H), 7.55-7.51 (m, 2H), 7.36-7.28 (m, 6H), 4.83 (s, 1H), 2.60-2.52 (m, 4H), 1.51- 1.43 (m, 2H).

Entry 3: 4-morpholino-4-phenylbut-2-yn-1-ol:

^1^H NMR (CDCl_3_, ppm): δ 7.55-7.49 (m, 2H), 7.45-7.28 (m, 3H), 4.60 (s, 1H), 4.38 (s, 2H), 3.73-3.68 (m, 4H), 2.64-2.60 (m, 4H)

Entry 4: 4-phenyl-4-(piperidin-1-yl)but-2-yn-1-ol:

^1^H NMR (250 MHz, CDCl_3_, ppm): δ 7.53-7.50 (m, 2H), 7.41-7.24 (m, 3H), 4.59 (s, 1H), 4.39 (s, 2H), 2.50-2.43 (m, 4H), 1.59-1.38 (m, 6H)

Entry 5: 4-(3-phenyl-1-(p-tolyl) prop-2-yn-1-yl)morpholine:

^1^H NMR (CDCl_3_, ppm): δ 7.55-7.50 (m, 5H), 7.35-7.18 (m, 4H), 4.78 (s, 1H), 3.77-3.74 (m, 4H), 2.68-2.63 (m, 4H), 2.37 (s, 3H).

Entry-6:1-(3-phenyl-1-p-tolylprop-2-ynyl)piperidine:

^1^H NMR (CDCl_3_, ppm): δ 7.57-7.53 (m, 4H), 7.35-7.30 (m, 3H), 7.19-7.14 (m, 2H), 4.79 (s, 1H), 3.64-3.54 (m, 4H), 2.35 (s, 3H), 1.67-1.56 (m, 4H), 1.51-1.42 (m, 2H).

Entry 7: 4-morpholino-4-(p-tolyl)but-2-yn-1-ol:

^1^H NMR (CDCl_3_, ppm): δ 7.53-7.46 (m, 2H), 7.40-7.35 (m, 2H), 4.58 (s, 1H), 4.37 (s, 2H), 3.72-3.68 (m, 4H), 2.62-2.58 (m, 4H), 2.37 (s, 3H).

Entry 8: 4‐(1‐(furan‐2‐yl)‐3‐phenylprop‐2‐ynyl)morpholine:

^1^H NMR (CDCl_3_, ppm): δ 7.57-7.48 (m, 3H), 7.37-7.30 (m, 3H), 6.60–6.35 (m, 2H), 4.92 (s, 1H), 3.85-3.73 (m, 4H), 2.74-2.62 (m, 4H).

Entry 9: 1-(1-(1-methyl-1H-pyrrol-2-yl)-3-phenylprop-2-yn-1-yl)piperidine:

^1^H NMR (CDCl_3_, ppm): δ 7.61-7.33 (m, 5H), 6.60 (d, 1H), 6.31-6.28 (m, 1H), 5.93 (d, 1H), 4.78 (s, 1H), 3.75 (s, 3H), 2.53-2.49 (m, 4H), 1.62-1.54 (m, 4H), 1.48-1.43 (m, 2H).

Entry 10: 4-(1-(1-methyl-1H-pyrrol-2-yl)-3-phenylprop-2-yn-1-yl)morpholine:

^1^H NMR (CDCl_3_, ppm): δ 7.63-7.36 (m, 5H), 6.61 (d, 1H), 6.31-6.26 (m, 1H), 5.95 (d, 1H), 4.82 (s, 1H), 3.81-3.68 (m, 7H), 2.76-2.59 (m, 4H).


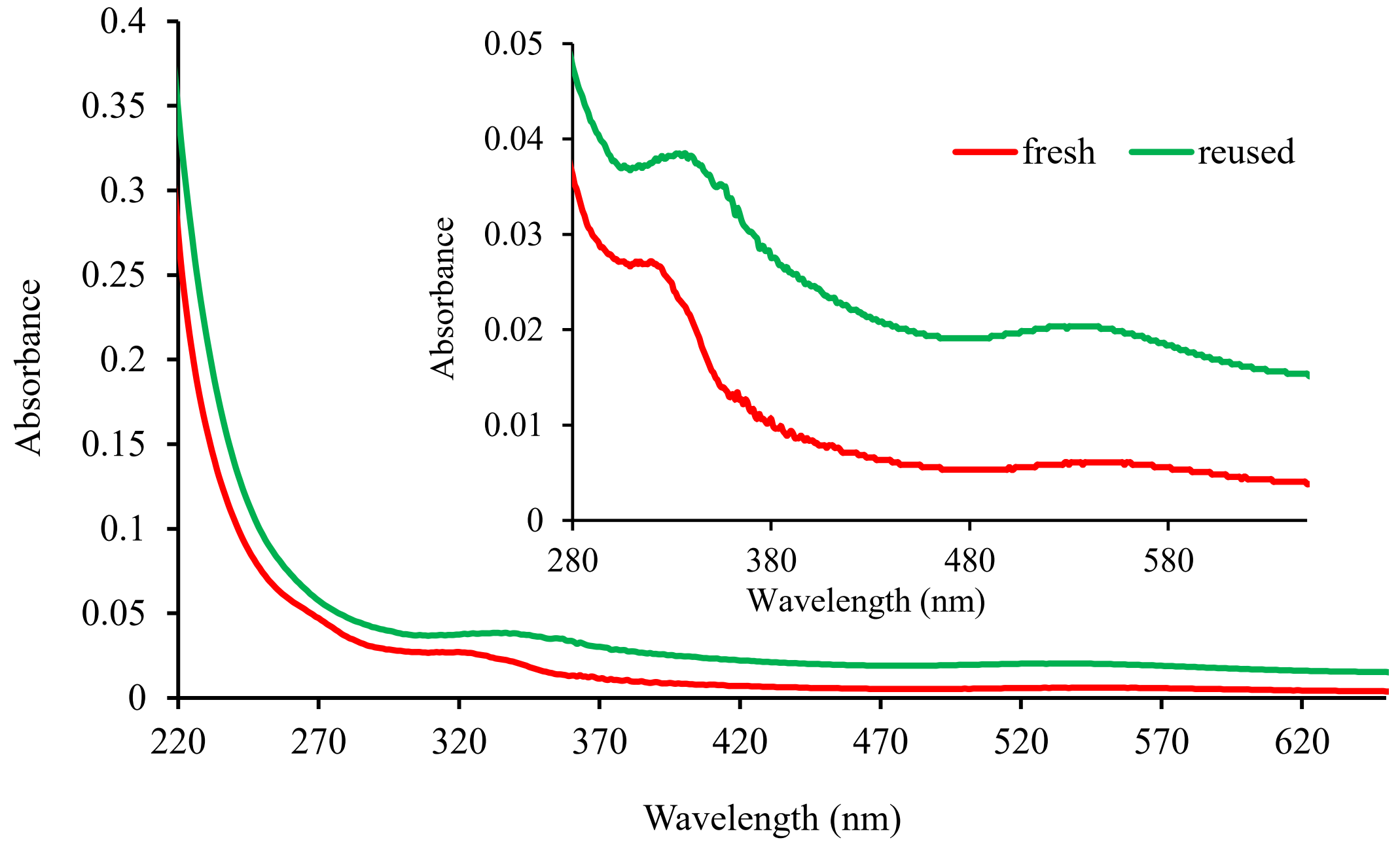


Fig. S4. UV-Vis spectra of fresh (red line) and reused (green line) complex **1** in acetonitrile medium

**References**

[1] CrysAlis CCD and CrysAlis Red 1.171.38.43, Rigaku Oxford Diffraction, Yarnton, UK. 2015.

[2] G.M. Sheldrick, SHELXT - Integrated space-group and crystal-structure determination. Acta Crystallogr. Sect. A: Found. Adv. **71** (2015) 3-8.

[3] G.M. Sheldrick, Crystal structure refinement with SHELXL. Acta Crystallogr. Sect. C: Cryst. Struct. Commun. **71** (2015) 3-8.

[4] K. Brandenburg, H. Putz, *DIAMOND* Version 3.0, Crystal Impact GbR, Bonn, Germany, 2006.
